# Interactive Effects of Apolipoprotein E ε4 and Triiodothyronine on Memory Performance in Patients With Subjective Cognitive Decline

**DOI:** 10.3389/fmed.2020.00298

**Published:** 2020-06-25

**Authors:** Jin San Lee, Yunsoo Soh, Hyug-Gi Kim, Kyung Mi Lee, Young Nam Kwon, Sung Sang Yoon, Key-Chung Park, Hak Young Rhee

**Affiliations:** ^1^Departments of Neurology, Kyung Hee University Hospital, Kyung Hee University College of Medicine, Seoul, South Korea; ^2^Departments of Rehabilitation Medicine, Kyung Hee University Hospital, Kyung Hee University College of Medicine, Seoul, South Korea; ^3^Departments of Radiology, Kyung Hee University Hospital, Kyung Hee University College of Medicine, Seoul, South Korea; ^4^Department of Medicine, Graduate School, Kyung Hee University, Seoul, South Korea; ^5^Department of Neurology, Kyung Hee University Hospital at Gangdong, Kyung Hee University College of Medicine, Seoul, South Korea

**Keywords:** thyroid hormone, apolipoprotein E, subjective cognitive decline, Alzheimer's disease, triiodothyronine

## Abstract

**Background:** The aim of the present study was to investigate the associations between thyroid hormones, cognitive performance, and apolipoprotein E (*APOE*) genotype in euthyroid patients with subjective cognitive decline (SCD).

**Methods:** We recruited 197 euthyroid patients that fulfilled the criteria for SCD. All participants were classified into *APOE* ε4 carriers and non-carriers based on the presence of the *APOE* ε4 allele. Patients with SCD who had the *APOE* ε2/ε4 genotype were excluded from the study. We then performed correlation and regression analyses to evaluate the associations between cognitive performance and thyroid hormones in *APOE* ε4 carriers and non-carriers.

**Results:** We found no significant differences in cognitive function between *APOE* ε4 carriers and non-carriers. However, higher levels of triiodothyronine (T3) were associated with better verbal memory performance (immediate and delayed recall tasks) in *APOE* ε4 carriers, whereas a negative association was found in *APOE* ε4 non-carriers. Furthermore, there was a significant interactive effect of *APOE* ε4 status and T3 levels on verbal memory performance (immediate and delayed recall tasks).

**Conclusions:** These findings suggest that in patients with SCD, T3 might have a protective effect on memory in those who are *APOE* ε4 carriers. The differential susceptibility hypothesis would thus support a gene-by-hormone crossover interaction between *APOE* ε4 allele and T3 in this study. Early identification and intervention of high-risk individuals for cognitive decline is important to establish new strategies for preventing dementia.

## Introduction

Thyroid hormones have been demonstrated to play an important role in cellular metabolism, growth, and differentiation of human organ systems. Thyrotropin-releasing hormone, produced by the hypothalamus, stimulates the release of thyroid-stimulating hormone (TSH) in the pituitary gland, which, in turn, induces the release of triiodothyronine (T3) and thyroxine (T4) in the thyroid gland (1). T4 is a major form of thyroid hormone in the blood, and has a longer half-life than T3. T4 is converted to the active T3 (three to four times more potent than T4), and this regulatory process is maintained by a neuroendocrine feedback mechanism in healthy individuals. Thyroid hormones are also essential for the development of the nervous system and play crucial roles in the maintenance of brain function (2). Common causes of reversible cognitive impairment include clinical hypothyroidism and hyperthyroidism (3, 4), and the thyroid function test has thus become a standard screening test in individuals who complain of cognitive decline (5).

Subjective cognitive decline (SCD) is the self-reported experience of worsening memory decline without objective cognitive deterioration (6). Previous studies have reported that SCD may represent the early symptomatic stage of Alzheimer's disease (AD) and related dementias (6, 7). SCD is part of a heterogeneous group of disorders, which includes preclinical AD and various conditions that can affect cognition such as depression and anxiety (6). Growing interest in strategies to maintain cognitive health in midlife has led many people who experience cognitive decline to visit a memory clinic (8, 9). However, lifestyle modifications, such as a healthy diet, adequate exercise, limiting alcohol, and abstaining from smoking are mainly recommended for most patients who have been diagnosed with SCD, unless other causes of cognitive deterioration are found.

Previous studies have reported interesting findings on the association between thyroid hormones and cognitive function in healthy euthyroid subjects. For instance, higher levels of T4 correlated positively with better general cognition in elderly men (10), and lower levels of T4 were related to a greater risk of cognitive worsening in elderly women (11)However, higher levels of free T3 were negatively correlated with executive functions in elderly women (1). In patients with mild cognitive impairment (MCI), the classically defined prodromal stage of dementia, higher levels of T3 were also negatively associated with cognitive performance across all cognitive domains (12), while lower levels of free T3 were associated with worse cognitive functioning in patients with coronary artery disease (13). While the investigation of thyroid hormones may be useful for assessments of cognitive performance in the elderly population, to date, knowledge regarding the relationship between thyroid hormones within the normal range and cognitive function in patients with SCD is limited.

Understanding the hormonal interrelationships that occur in SCD can provide opportunities for earlier interventions in patients who are progressing to MCI or dementia. The objective of this study was to investigate the relationship between thyroid hormones as well as TSH and cognitive performance in euthyroid patients with SCD. Specifically, considering that the apolipoprotein E (*APOE*) ε4 allele is not only a genetic risk factor for sporadic AD but also for earlier stages such as MCI or even SCD (14, 15), we hypothesized that thyroid hormones as well as TSH may have distinct effects on cognitive performance in participants depending on their *APOE* ε4 status.

## Materials and Methods

### Study Participants

**Figure 1:**
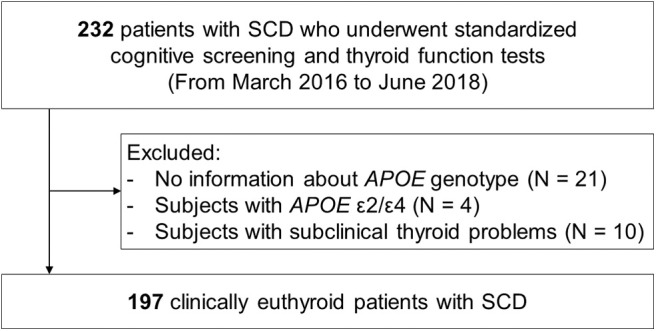
Flow chart of the study participants. SCD, subjective cognitive decline; MRI, magnetic resonance imaging; *APOE*, apolipoprotein E.

The flow chart of the study participants is presented in Figure 1. We consecutively recruited 232 patients with SCD at age 50 or older from the Memory Clinic at Kyung Hee University Hospital (Seoul, Korea) from March 2016 to June 2018, in line with the following criteria (16): (1) subjective memory complaints by patients or caregivers, (2) no objective cognitive dysfunction in any cognitive domain in detailed neuropsychological tests, and (3) no dementia. All participants underwent a standardized diagnostic assessment protocol for cognitive impairment and dementia including high-resolution 3.0T magnetic resonance imaging (MRI) as well as detailed neuropsychological tests. Brain MRI confirmed the absence of structural lesions including cerebral hemorrhage or infarction, hippocampal sclerosis, brain tumors, traumatic encephalomalacia, and vascular malformation. The exclusion criteria included a history of thyroid axis disorders, thyroid hormone replacement therapy, psychological disease, stroke, brain surgery, seizure, head trauma, severe cerebral white matter hyperintensities (deep white matter ≥ 25 mm, and caps or band ≥ 10 mm), other medication that could interfere with thyroid hormone metabolism (such as amiodarone), and current systemic medical diseases that could affect cognition.

Laboratory tests were conducted to exclude other causes of cognitive impairment, and included thyroid function tests (T3, free T4 [fT4], and TSH), a metabolite profile, vitamin B12 and folate levels, complete blood counts, blood chemistry, and syphilis serology. *APOE* genotyping was performed in 211 (90.9%) of the 232 participants. We excluded four patients who had the *APOE* ε2/ε4 genotype from the study, since the putative opposing effects of the ε4 and ε2 alleles could result in some confusion in the interpretation of results (17, 18). All study participants were clinically euthyroid, but 10 participants showed subclinical thyroid problems in endocrinological assessments, and these subjects were also excluded from the study. The final sample size was 197.

### Standard Protocol Approval, Registration, and Patient Consent

Written informed consent was obtained from all participants before inclusion in the study. The study was approved by the Institutional Review Board (IRB) of Kyung Hee University Hospital (IRB file number: 2018-01-023). All procedures were carried out in accordance with approved guidelines.

### Neuropsychological Testing and Clinical Assessments

All participants underwent detailed neuropsychological tests using the standardized Seoul Neuropsychological Screening Battery (19, 20). The battery contains tests for attention (the Digit Span Forward and Backward), language (the Korean version of the Boston Naming Test [K-BNT]), visuospatial function (the Rey-Osterrieth Complex Figure Test [RCFT]; copying), verbal and visual memory (the Seoul Verbal Learning Test [SVLT] and RCFT; immediate and 20-min delayed recall, and recognition), and frontal/executive function (the phonemic and semantic Controlled Oral Word Association Test [COWAT] and a Stroop Test; word and color reading). Cognitive functions associated with each neuropsychological test are presented in Table 1. Age- and education-specific norms for each test based on 447 cognitively normal individuals were used for comparison. Z-scores lower than −1.0 standard deviation (SD) of the age- and education-adjusted norms were considered abnormal. We also used the Mini-Mental Status Examination (MMSE), the Clinical Dementia Rating, the Clinical Dementia Rating Sum of Boxes, and the Geriatric Depression Scale.

**Table 1:** Brief descriptions of the neuropsychological tests and related cognitive functions.

**Cognitive domains and neuropsychological tests**	**Related cognitive functions**
**Attention**	
Digit span forward	Attention efficiency and capacity
Digit span backward	Working memory, attention
**Language**	
K-BNT	Confrontation naming, semantic representation
**Visuospatial**	
RCFT copy	Visuo-perceptive and visuo-constructive functions
**Verbal memory**	
SVLT immediate recall	Learning ability (verbal memory)
SVLT delayed recall	Consolidation ability (verbal memory)
SVLT recognition	Retrieval ability (verbal memory)
**Visual memory**	
RCFT immediate recall	Learning ability (visual memory)
RCFT delayed recall	Consolidation ability (visual memory)
RCFT recognition	Retrieval ability (visual memory)
**Frontal/executive**	
COWAT phonemic total	Frontal executive function, language function
Stroop color reading	Selective attention, cognitive flexibility
**Global**	
MMSE	Overall measure of global cognitive functioning

*K-BNT, Korean version of the boston naming test; RCFT, Rey-osterrieth complex figure test; SVLT, Seoul verbal learning test; COWAT, Controlled oral word association test; MMSE, Mini-mental state examination*.

### *APOE* Genotyping

Genomic DNA was extracted from peripheral blood leukocytes using the QIAamp DSP DNA Mini Kit following the manufacturer's instructions (QIAGEN GmbH, Hilden, Germany). Two single nucleotide polymorphisms (rs429358 for codon 112 and rs7412 for codon 158) in the *APOE* gene were genotyped using LG AdvanSure^TM^ apoE Genotyping real-time PCR (LG Lifescience, Korea) on a SLAN real-time PCR Detection System (LG Lifescience, Korea) according to the manufacturer's instructions. Subjects with at least one *APOE* ε4 allele were identified as ε4 carriers. In addition, subjects with ε2/ε2, ε2/ε3, and ε3/ε3 alleles were identified as ε4 non-carriers.

### Thyroid Function Tests

Serum levels of total T3, fT4, and TSH were evaluated with a chemiluminescence immunoassay using the STRATEC SR 300 analyzer (Brahms, Berlin, Germany). According to our laboratory-verified reference ranges, the normal serum T3, fT4, and TSH intervals were 76–170, 0.9–1.8, and 0.5–4.5 μIU/mL, respectively.

### Statistical Analyses

Continuous variables were presented as means ± SD and were compared using the Student's *t*-test. Categorical variables were compared using a Chi-square test or Fisher's exact test. For comparisons of neuropsychological performance between *APOE* ε4 carriers and non-carriers, we used age- and education-specific Z-scores. To evaluate correlations between the results of neuropsychological tests and thyroid hormones as well as TSH according to *APOE* ε4 status, bivariate relationships were calculated using Pearson's correlation coefficient. Multiple linear regression was performed using sex, vascular risk factors (hypertension, diabetes mellitus, hyperlipidemia, cardiovascular disease, and history of stroke), *APOE* ε4 status, thyroid hormones including TSH, and an interaction term (thyroid hormones including TSH by *APOE* ε4 status) as independent variables. All tests were two-tailed, and statistical significance was set at *p* < 0.05. The Statistical Package for the Social Sciences (SPSS) version 20.0 (SPSS Inc., Chicago, IL, USA) was used to perform all statistical analyses.

## Results

### Demographic and Clinical Characteristics

Demographic and clinical characteristics of the study participants are presented in Table 2. The mean age of our participants was 65.5 years, and 142 (72.1%) were female. We identified 49 (24.9%) *APOE* ε4 carriers, and there were no significant differences in demographics between the carriers and the non-carriers except for education level. Furthermore, there were no significant differences in thyroid hormones and TSH levels between the two groups.

**Table 2:** Demographic and clinical characteristics of the study participants.

	**Total**	***APOE*** **ε4 status**		***p*-value**
		**Non-carriers**	**Carriers**	
*N*	197 (100.0)	148 (75.1)	49 (24.9)	
Age, years	65.5 (8.3)	65.5 (8.2)	65.2 (8.7)	0.857
Age range, years	50–88	51–88	50–85	
Women	142 (72.1)	110 (74.3)	32 (65.3)	0.223
Education, years	11.2 (4.6)	10.7 (4.9)	12.5 (3.4)	0.005
**Vascular risk factors**				
Hypertension	75 (38.1)	59 (39.9)	16 (32.7)	0.368
DM	27 (13.7)	20 (13.5)	7 (14.3)	0.892
Hyperlipidemia	68 (34.5)	49 (33.1)	19 (38.8)	0.470
Cardiovascular disease	28 (14.2)	20 (13.5)	8 (16.3)	0.625
History of stroke	1 (0.5)	1 (0.7)	0 (0.0)	0.564
***APOE*** **genotype**				
ε2 carriers	16 (8.1)	16 (10.8)		
ε3/ε3	132 (67.0)	132 (89.2)		
ε4 carriers	49 (24.9)		49 (100.0)	
**Thyroid hormones**				
T3, ng/dL	104.7 (17.1)	104.1 (17.4)	106.4 (16.0)	0.408
Range	76.1–167.9	76.1–167.9	81.2–161.1	
fT4, ng/dL	1.3 (0.2)	1.3 (0.2)	1.2 (0.2)	0.587
Range	0.9–1.8	0.9–1.8	0.9–1.8	
TSH, μIU/mL	2.3 (1.2)	2.3 (1.2)	2.5 (1.3)	0.400
Range	0.5–4.5	0.5–4.0	0.7–4.5	

*Values are mean (SD) or N (%). Chi-square and Student's t-tests were performed for comparisons of variables between APOE ε4 carriers and non-carriers. N, number; SD, standard deviation; APOE, apolipoprotein E; DM, diabetes mellitus; T3, triiodothyronine; fT4, free thyroxine; TSH, thyroid-stimulating hormone*.

### Comparisons of Neuropsychological Performances

Table 3 shows comparisons of neuropsychological performance between *APOE* ε4 carriers and non-carriers among our patients with SCD. Although the mean Z-scores of neuropsychological tests were higher in non-carriers than in carriers, except for the Digit Span Forward Test, there were no statistically significant differences between the two groups.

**Table 3:** Comparisons of neuropsychological performance between *APOE* ε4 carriers and non-carriers in patients with SCD.

	**Total**	***APOE*** **ε4 status**		***p*-value**
		**Non-carriers**	**Carriers**	
**Attention**				
Digit span forward	0.4 (1.1)	0.3 (1.1)	0.6 (1.0)	0.146
Digit span backward	0.6 (1.3)	0.7 (1.4)	0.4 (1.1)	0.218
**Language**				
K-BNT	0.6 (0.7)	0.6 (0.7)	0.6 (0.7)	0.909
**Visuospatial function**				
RCFT copy	0.5 (0.6)	0.5 (0.6)	0.4 (0.7)	0.152
**Verbal memory**				
SVLT immediate recall	0.7 (1.4)	0.8 (1.6)	0.4 (0.9)	0.166
SVLT delayed recall	0.6 (1.0)	0.6 (1.1)	0.6 (0.9)	0.734
SVLT recognition	0.5 (0.8)	0.5 (0.8)	0.4 (0.8)	0.618
**Visual memory**				
RCFT immediate recall	0.4 (0.9)	0.5 (0.9)	0.4 (0.8)	0.421
RCFT delayed recall	0.4 (0.8)	0.4 (0.8)	0.3 (0.7)	0.610
RCFT recognition	0.5 (1.1)	0.6 (1.0)	0.4 (1.3)	0.236
**Frontal/executive function**				
COWAT phonemic total	0.8 (1.2)	0.8 (1.2)	0.8 (1.1)	0.947
Stroop color reading	0.6 (0.7)	0.6 (0.7)	0.5 (0.7)	0.766
**Global**				
MMSE	0.5 (0.7)	0.6 (0.6)	0.4 (0.7)	0.074

*Age- and education-specific Z-scores were used for comparisons of neuropsychological performance between APOE ε4 carriers and non-carriers in patients with SCD. APOE, apolipoprotein E; SCD, subjective cognitive decline; K-BNT, Korean version of the boston naming test; RCFT, Rey-osterrieth complex figure test; SVLT, Seoul verbal learning test; COWAT, Controlled oral word association test; MMSE, Mini-mental state examination*.

### Correlation Between Neuropsychological Performance and Thyroid Hormones as Well as TSH

The results of the correlation analyses between neuropsychological performance and thyroid hormones as well as TSH are presented in Table 4. There was a negative correlation between T3 levels and Digit Span Forward and RCFT recognition task Z-scores in our patients with SCD. Levels of T3 and TSH correlated negatively with the Z-score of the Digit Span Forward task in *APOE* ε4 carriers. Levels of T3 correlated negatively with the Z-scores of the SVLT immediate and delayed recall as well as the RCFT recognition task in *APOE* ε4 non-carriers, while in *APOE* ε4 carriers, levels of T3 correlated positively with the Z-scores of the SVLT immediate and delayed recall tasks. In addition, there was a positive overall correlation between fT4 levels and Z-scores of the COWAT phonemic total task and the MMSE in our patients with SCD. Specifically, levels of fT4 correlated positively with the Z-score of the COWAT phonemic total task in *APOE* ε4 non-carriers. In *APOE* ε4 carriers, levels of TSH correlated negatively with the Z-score of the Digit Span Forward task.

**Table 4:** Correlation coefficient (r) between neuropsychological performance and thyroid hormone levels as well as TSH according to *APOE* ε4 status.

	**T3**			**fT4**			**TSH**		
	**Total**	**Non-carriers**	**Carriers**	**Total**	**Non-carriers**	**Carriers**	**Total**	**Non-carriers**	**Carriers**
**Attention**									
Digit span forward	−0.174^*^	−0.132	−0.368^*^	−0.085	−0.085	−0.018	−0.129	−0.064	−0.351^*^
Digit span backward	0.025	0.039	−0.004	−0.043	−0.080	0.028	−0.023	0.003	−0.087
**Language**									
K-BNT	0.071	0.078	0.045	0.049	<0.001	0.201	0.030	−0.005	0.129
**Visuospatial**									
RCFT copy	−0.001	−0.066	0.208	0.113	0.134	0.021	0.120	0.072	0.253
**Verbal memory**									
SVLT immediate recall	−0.055	−0.119^*^	0.352^*^	0.073	0.063	0.056	0.055	0.037	0.187
SVLT delayed recall	−0.081	−0.176^*^	0.284^*^	0.079	0.085	0.059	0.037	0.023	0.088
SVLT recognition	−0.023	−0.080	0.188	0.014	−0.024	0.140	0.030	0.040	0.011
**Visual memory**									
RCFT immediate recall	−0.102	−0.122	−0.013	−0.031	−0.027	−0.091	−0.030	−0.067	0.092
RCFT delayed recall	−0.073	−0.101	0.038	0.007	0.051	−0.182	0.007	−0.057	0.202
RCFT recognition	−0.198^*^	−0.189^*^	−0.217	−0.023	−0.058	0.011	−0.022	0.034	−0.118
**Frontal/executive**									
COWAT phonemic total	−0.056	−0.046	−0.097	0.199^*^	0.209^*^	0.187	−0.036	−0.026	−0.066
Stroop color reading	0.002	−0.035	0.125	0.016	0.016	−0.005	0.001	0.029	−0.067
**Global**									
MMSE	0.002	−0.033	0.128	0.147^*^	0.140	0.121	−0.122	−0.062	−0.231

*Pearson's correlation coefficients were calculated using age- and education-specific Z-scores*.

* *Indicates p < 0.05. APOE, apolipoprotein E; SCD, subjective cognitive decline; T3, triiodothyronine; fT4, free thyroxine; TSH, thyroid-stimulating hormone; K-BNT, Korean version of the boston naming test; RCFT, Rey-osterrieth complex figure test; SVLT, Seoul verbal learning test; COWAT, Controlled oral word association test; MMSE, Mini-mental state examination*.

### Interaction Effect of *APOE* ε4 Status and T3 Level on Neuropsychological Performance

**Figure 2:**
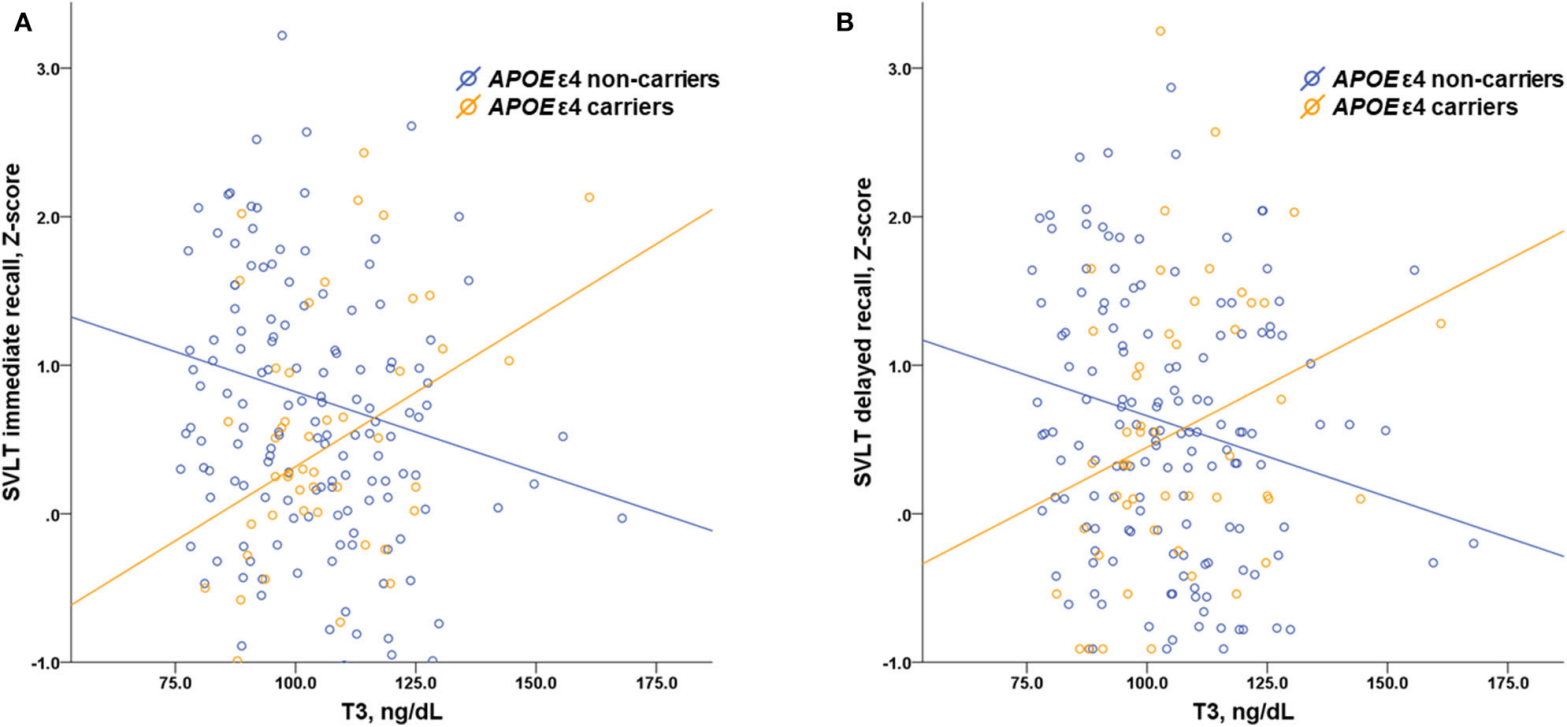
Scatter plots investigating the relation between memory performance and serum T3 levels in patients with SCD according to *APOE* ε4 status showing interaction effects of *APOE* ε4 status and T3 level on the results of **(A)** the SVLT immediate recall (*p* = 0.032) and **(B)** the SVLT delayed recall (*p* = 0.004) task. T3, triiodothyronine; SCD, subjective cognitive decline; *APOE*, apolipoprotein E; SVLT, Seoul verbal learning test.

Multiple linear regressions were performed to evaluate the interactive effects of *APOE* ε4 status and thyroid hormones as well as TSH on neuropsychological performance (Table 5). There were significant interactive effects of *APOE* ε4 status and T3 level on the RCFT copy (*p* = 0.045), SVLT immediate (*p* = 0.032), and delayed recall (*p* = 0.004) tasks, suggesting that higher levels of T3 were associated with better memory performance in *APOE* ε4 carriers. Figure 2 shows scatter plots investigating the relation between neuropsychological performance and T3 levels in our patients with SCD according to *APOE* ε4 status.

**Table 5:** Interaction effects of *APOE* ε4 status and thyroid hormone levels as well as TSH on neuropsychological performance.

	T3^*^APOE **ε4**			fT4^*^APOE **ε4**			TSH^*^APOE **ε4**		
	**B**	**SE**	***p***	**B**	**SE**	***p***	**B**	**SE**	***p***
**Attention**									
Digit span forward	−0.014	0.011	0.215	0.280	0.757	0.711	−0.151	0.121	0.213
Digit span backward	−0.003	0.014	0.840	0.611	0.952	0.522	−0.034	0.152	0.822
**Language**									
K-BNT	<0.001	0.008	0.985	0.543	0.519	0.296	0.083	0.083	0.318
**Visuospatial**									
RCFT copy	0.012	0.006	0.045^*^	−0.291	0.432	0.502	0.078	0.068	0.251
**Verbal memory**									
SVLT immediate recall	0.032	0.015	0.032^*^	−0.161	1.046	0.878	0.045	0.164	0.783
SVLT delayed recall	0.030	0.010	0.004^*^	−0.269	0.731	0.713	0.019	0.115	0.869
SVLT recognition	0.013	0.008	0.097	0.469	0.559	0.402	−0.060	0.088	0.499
**Visual memory**									
RCFT immediate recall	0.006	0.009	0.483	−0.240	0.643	0.709	0.115	0.101	0.256
RCFT delayed recall	0.007	0.008	0.423	−0.750	0.568	0.188	0.137	0.090	0.128
RCFT recognition	−0.009	0.011	0.440	0.408	0.792	0.607	−0.135	0.126	0.285
**Frontal/executive**									
COWAT phonemic total	−0.004	0.012	0.741	−0.265	0.836	0.752	−0.028	0.135	0.835
Stroop color reading	0.008	0.007	0.275	−0.167	0.495	0.736	−0.061	0.079	0.437
**Global**									
MMSE	0.006	0.007	0.358	−0.059	0.480	0.902	−0.093	0.076	0.222

*Multiple linear regression was performed using sex, vascular risk factors (hypertension, diabetes mellitus, hyperlipidemia, cardiovascular disease, and history of stroke), APOE ε4 status, thyroid hormones including TSH, and an interaction term (thyroid hormones including TSH by APOE ε4 status) as independent variables. Age- and education-specific Z-scores were used as dependent variables*.

* *Indicates p < 0.05. APOE, apolipoprotein E; SCD, subjective cognitive decline; T3, triiodothyronine; fT4, free thyroxine; TSH, thyroid-stimulating hormone; B, β value; SE, standard error; K-BNT, Korean version of the boston naming test; RCFT, Rey-osterrieth complex figure test; SVLT, Seoul verbal learning test; COWAT, Controlled oral word association test; MMSE, Mini-mental state examination*.

## Discussion

In this study, we investigated the effect of thyroid hormones as well as TSH on cognitive performance in *APOE* ε4 carriers and non-carriers among euthyroid patients with SCD. The key findings of our study are that levels of T3 correlated positively with memory performance in *APOE* ε4 carriers, whereas a negative correlation was found in *APOE* ε4 non-carriers. Moreover, there was a significant interactive effect of *APOE* ε4 status and T3 level on memory performance. This suggests that T3 has a protective effect on memory in *APOE* ε4 carriers with SCD, which might represent a risk group for cognitive deterioration and development of AD.

Individuals with SCD have generally been regarded as the “worried well,” used to describe individuals who are at risk of developing disease, given the lack of objective evidence of cognitive impairment. However, previous studies suggest that, for substantial numbers of individuals with SCD, self-reported experience of worsening memory decline may indeed herald the development of cognitive decline to dementia (16, 21). Apolipoprotein E, on the other hand, is a plasma lipoprotein that has functions in Aβ clearance (22). *APOE* ε4 has been well-established as an important risk factor for developing AD (23), whereas *APOE* ε2 reduces the risk of AD (24). In general, *APOE* ε4 has been an established risk factor for memory decline, despite ongoing cognitively normal status (25–27). In a recent meta-analysis, the presence of *APOE* ε4 increased the risk of conversion to AD from 0.78% per year among non-carriers to 3.24% per year among carriers among patients with SCD (15). Therefore, *APOE* ε4 carriers with SCD have an additional risk for dementia (25, 28).

Current evidence have allowed for a shift in the definition of AD from a syndromal to a biological construct, based on biomarkers that are proxies of pathology (29). However, little is known about mechanisms underlying the disease progression at its early stages, such as SCD. To date, various clinical trials focusing on multimodal interventions (nutritional, physical, cognitive, and medical) have attempted to prevent the progression of dementia in patients with SCD. However, the available evidence with regards to lifestyle interventions for SCD is limited (30). Our finding that T3 may have a protective effect on memory in *APOE* ε4 carriers with SCD has clinical significance in terms of prevention of cognitive deterioration and dementia. Although not much research on the supplementation of thyroid hormones in patients with SCD has been reported to date, we speculate that supplementing T3 in *APOE* ε4 carriers with SCD might help prevent cognitive decline.

Indeed, we have found some evidence to support our speculation in a few previous studies on several neuropsychological diseases: in depressed patients with normal levels of thyroid hormones, the addition of T3 to antidepressant drugs had some benefit in the treatment of both manic and depressed phases of mood disorders (31); application of T3 orally improved performance on a verbal fluency task in healthy subjects (32); and partial substitution of T3 for T4 led to improved neuropsychological performance and mood in patients with hypothyroidism (33). However, these findings need to be interpreted with caution, since these results are derived from groups with other diseases and small sample sizes. Contrary to our suggestion, a previous report demonstrated that the use of thyroid drugs was associated with the incidence of AD dementia (34). Thyroid hormone therapy with levothyroxine also provided no benefit with regard to executive cognitive function in older persons with subclinical hypothyroidism (35). Moreover, elderly subjects who had high levels of thyrotropin, above the normal range, were found to have an elongated life span (36).

The detrimental effect of T3 on memory in *APOE* ε4 non-carriers could be explained by several reports of a potential direct action of T3 on cognitive performance. Aggravation of the cholinergic deficit and related cognitive dysfunction observed in patients with AD has been suggested to be due to thyroid hormone-induced depletion of acetylcholine (37). Thyroid hormone-induced oxidative damage and reduced antioxidative defense enzyme levels have been associated with progressive neurodegeneration (38). However, there is little biological evidence for the protective effect of T3 on memory in *APOE* ε4 carriers with SCD. Thyroid hormones modulate gene expression and intracellular signal transduction by regulating the synthesis of enzymes necessary for the production of neurotransmitters (2). We suggest that the differential susceptibility hypothesis (39)—genetic factors that are supposed to confer vulnerability may lead to differential susceptibility to both the negative and positive effects of some other factor—supports a gene-by-hormone crossover interaction between the *APOE* ε4 allele and T3 in this study. In other words, *APOE* ε4 carriers with higher levels of T3 (beneficial circumstances) may function better than *APOE* ε4 non-carriers, which is consistent with the results from a previous study reporting on testosterone (40). Alternatively, as the *APOE* ε4 allele has been reported to be positively associated with hypothyroidism (41), this mechanism might also be related to compensation.

The strength of our study is the sample size that enabled us to conduct substantial statistical analyses. However, there are some limitations. First, our study has a cross-sectional design, which prevents us from making claims of causality. Second, the study participants were recruited from a memory disorder clinic, and the sample might thus not be representative of the general population. Third, the interpretation of our findings is limited since serum concentrations of thyroid hormones do not accurately reflect the metabolism of thyroid hormones in the brain. Fourth, although we confirmed that our participants had no crucial metabolic problems, it is possible that other factors or metabolic pathways influenced the effects of thyroid hormones on cognitive function we observed. Finally, although sex is regarded to be an important parameter in estimating the risk of AD or thyroid disease (42), we did not perform analyses according to sex, due to the relatively small number of men in our sample. Nevertheless, our findings provide important insights that help our understanding of the associations between thyroid hormones, cognitive performance, and *APOE* genotypes in patients with SCD. Early identification of high-risk individuals and interventions for cognitive decline are important in the quest to establish new strategies for preventing dementia, in keeping with the paradigm shift in focus from AD dementia to preclinical AD in the development of therapeutic interventions. However, further evidence for the supplementation of T3 in *APOE* ε4 carriers with SCD to prevent cognitive decline can only be gathered from a well-designed, randomized clinical trial.

## Data Availability Statement

The datasets generated for this study are available on request to the corresponding author.

## Ethics Statement

The studies involving human participants were reviewed and approved by institutional review board at the Kyung Hee university hospital. The patients/participants provided their written informed consent to participate in this study.

## Author Contributions

JL and HR: conception and design of the study and final approval of the manuscript. JL, SY, K-CP, and HR: acquisition of data. JL, YS, H-GK, KL, YK, SY, K-CP, and HR: analysis and interpretation of the data. JL, YK, and HR: drafting and revising the manuscript for content.

## Conflict of Interest

The authors declare that the research was conducted in the absence of any commercial or financial relationships that could be construed as a potential conflict of interest.
